# A Novel Targeted Conjugate for Photodynamic Therapy of HER2-Positive Cancer

**DOI:** 10.32607/actanaturae.27891

**Published:** 2026

**Authors:** I. P. Sharychev, A. A. Prach, E. V. Plotnikov, M. S. Larkina, P. V. Petunin, M. V. Belousov, E. V. Konovalova, A. A. Shulga, S. M. Deev

**Affiliations:** National Research Tomsk Polytechnic University, Tomsk, 634050 Russia; Laboratory of Molecular Cancer Therapy, Cancer Research Institute, Tomsk National Research Medical Center, Russian Academy of Sciences, Tomsk, 634028 Russia; Siberian State Medical University, Tomsk, 634050 Russia; Mental Health Research Institute of the Tomsk National Research Medical Center, Tomsk, 634014 Russia; Molecular Immunology Laboratory, Shemyakin–Ovchinnikov Institute of Bioorganic Chemistry, Russian Academy of Sciences, Moscow, 117997 Russia

**Keywords:** targeted photodynamic therapy, IR700, DARPin, phototoxicity, HER2

## Abstract

Low selectivity against cancer cells remains one of the key challenges of many modern photosensitizers. To enhance the selectivity of the action and the effectiveness of tumor therapy, a conjugate was developed comprising a photoactive compound, the water-soluble silicon phthalocyanine complex (IR700, IRDye 700DX NHS ester), and the targeted molecule DARPin G3-ABD, which is designed to target human epidermal growth factor receptor type 2 (HER2). The phototoxicity of the individual substance IR700 and the DARPin G3-ABD-IR700 conjugate was tried on tumor cell cultures with different HER2 expression levels (SKBR-3, SKOV-3, BT-474, and PC-3). The cells were irradiated using LED diodes with a wavelength of 690 nm and a luminous flux density of 0.5 W/cm^2^. Cell viability after irradiation and following all the experimental procedures was evaluated using the standard MTT assay. The developed conjugate proved to specifically bind to HER2 receptors. The combination of high photosensitizing activity and selectivity of action against tumor cells makes the DARPin G3-ABD-IR700 conjugate a promising targeted agent for photodynamic therapy.

## INTRODUCTION

Cancer remains one of the leading causes of death worldwide [1]. Forecasting models predict that by 2050, cancer incidence and mortality will increase by 60.7% and 74.5%, respectively [2]. This unfavorable trend underscores the need to develop novel approaches to improving the efficacy of tumor therapy.

Modern clinical oncology employs a broad range of antitumor modalities, with surgery, chemotherapy, and radiotherapy serving as standard treatments. However, these approaches are often accompanied by adverse side effects that substantially impair patients’ quality of life [3]. In recent years, considerable focus has been directed on minimally invasive methods for the treatment of neoplastic diseases, among which photodynamic therapy (PDT) is a promising approach [4].

The method is based on a dynamic interaction between a photosensitizer (PS), light of a specific wavelength, and, in most cases, molecular oxygen [5]. The PS accumulates in tumor tissues and is transferred from the ground state to an excited state upon exposure to light or laser radiation. Photochemical reactions generate free radicals and radical ions (reactive oxygen species causing oxidative damage to cancer cells) [6].

Irradiation in the so-called therapeutic window (600–850 nm) is predominantly used in optimizing photodynamic action, since near-infrared light has a high capacity to penetrate biological tissues [7, 8].

A key limitation of most conventional PSs is their low tumor selectivity [6]. A promising solution is to develop specific targeted agents by conjugating PSs to antibodies exhibiting an affinity for certain molecular targets [9, 10]. Human epidermal growth factor receptor 2 (HER2), a transmembrane glycoprotein belonging to the tyrosine kinase family, is a common tumor marker. HER2 plays a key role in the pathogenesis of several cancer types, including breast, ovarian, and gastric cancer, by promoting an uncontrolled growth of tumor cells [11]. Preclinical [12, 13] and clinical (phase 1/2a [14]) studies attest to the high efficacy of therapeutic conjugates for PDT. However, the large size of monoclonal antibodies (150 kDa) limits their ability to penetrate biological tissues and slows down their elimination from the body [15]. These limitations can be overcome by developing small targeting molecules (6–7 kDa) with high affinity for HER2. Therapeutic PS conjugates with several small targeting molecules have been investigated, including affibodies for *in vivo* tumor imaging [16] and peptidomimetics conjugated to photosensitive compounds [17, 18].

A similar approach was implemented in this study to create a novel targeted conjugate using highly specific genetically engineered proteins based on ankyrin repeats (DARPins) [19, 20]. A silicon phthalocyanine complex in the form of a succinimidyl ester (IRDye 700DX NHS ester), which had previously showed potential for PDT, was used as the photoactive component [9, 18, 21]. The DARPin G3-ABD fusion protein, composed of a scaffold protein (DARPin G3) [15] and an albumin-binding domain (ABD), is the targeting component of the conjugate, which selectively binds HER2 [22]. Such a construct has previously been reported to ensure high tumor-targeting specificity [23, 24].

Therefore, this study aimed to develop and evaluate the *in vitro* efficacy of a novel targeted agent for photodynamic therapy of HER2-positive cancer.

## EXPERIMENTAL

**Reagents**

IR700 (IRDye 700DX NHS ester, C_74_H_96_N_12_Na_4_O_27_S_6_Si_3_, 1954.2 g/mol) is a water-soluble silicon phthalocyanine dye containing an N-hydroxysuccinimide ester group (Shanghai Tachizaki Biomedical Research Center, China). Its principal spectral characteristics are as follows: absorption maximum, 689 nm; emission maximum, 700 nm; molar extinction coefficient, 165.0 M^-1^∙cm^-1^ [25].

The targeting protein molecule DARPin G3-ABD (21,253.83 g/mol) was produced biotechnologically at the Institute of Bioorganic Chemistry of the Russian Academy of Sciences (Moscow, Russia), as described previously [23, 24].

**Synthesis of the DARPin G3-ABD-IR700 conjugate**

The synthesis procedure for the targeted conjugate comprised two key stages: the preparative stage aimed at protein reduction and dimerization prevention, followed by direct conjugation of DARPin G3-ABD to IR700. Degassed phosphate-buffered saline (0.02 M, pH 7.4) was used as a solvent to prepare reagent solutions.

At the first stage, a dithiothreitol solution (20 mg/mL, 11 μL, 3.5 μmol) was added to the DARPin G3-ABD solution (9.07 mg/mL, 110 μL, 47 nmol). The resulting mixture was vortexed and incubated at 40°C for 1 h. A chloroacetamide solution (20 mg/mL, 54 μL, 11.6 μmol) was then added. The solution was incubated at room temperature for 30 min. The protein was purified using NAP-5 columns (GE Healthcare, United Kingdom), then pre-equilibrated, and eluted, using phosphate-buffered saline (pH 8.5). The protein concentration was measured spectrophotometrically at 280 nm using a NanoDrop OneC spectrophotometer (Thermo Fisher Scientific, USA).

DARPin G3-ABD was then conjugated to IR700. The IR700 solution in DMSO (5 mg/mL, 60 μL, 100.8 nmol) was added to the isolated DARPin G3-ABD (450 μL, 33.6 nmol), followed by incubation at 40°C for 1 h and then overnight at 4°C. The resulting DARPin G3-ABD-IR700 conjugate was purified using NAP-5 columns. The protein concentration was determined spectrophotometrically at 280 and 690 nm.

The purity of the resulting conjugate was determined by polyacrylamide gel electrophoresis [26].

**Cell cultures**

Standardized cell cultures with different HER2 expression levels were used as biological models for *in vitro* studies, including human breast cancer cells (SKBR-3 and BT-474), human ovarian cancer cells (SKOV-3), and human prostate cancer cells (PC-3) (PrimeBioMed LLC, Moscow, Russia).

The cells were cultured in a standard DMEM/F-12 medium (DMEM, Gibco, Billings, MT, USA) supplemented with a penicillin–streptomycin antibiotic mixture, 10% fetal bovine serum, and L-glutamine (PanEco LLC, Russia). The cells were maintained in a CO_2_ incubator at 37°C in a humidified atmosphere containing 5% CO_2_.

**Light irradiation of cell cultures**

Photodynamic treatment of tumor cells was performed using the experimental setup shown in *Fig. 1*.

**Fig. 1:**
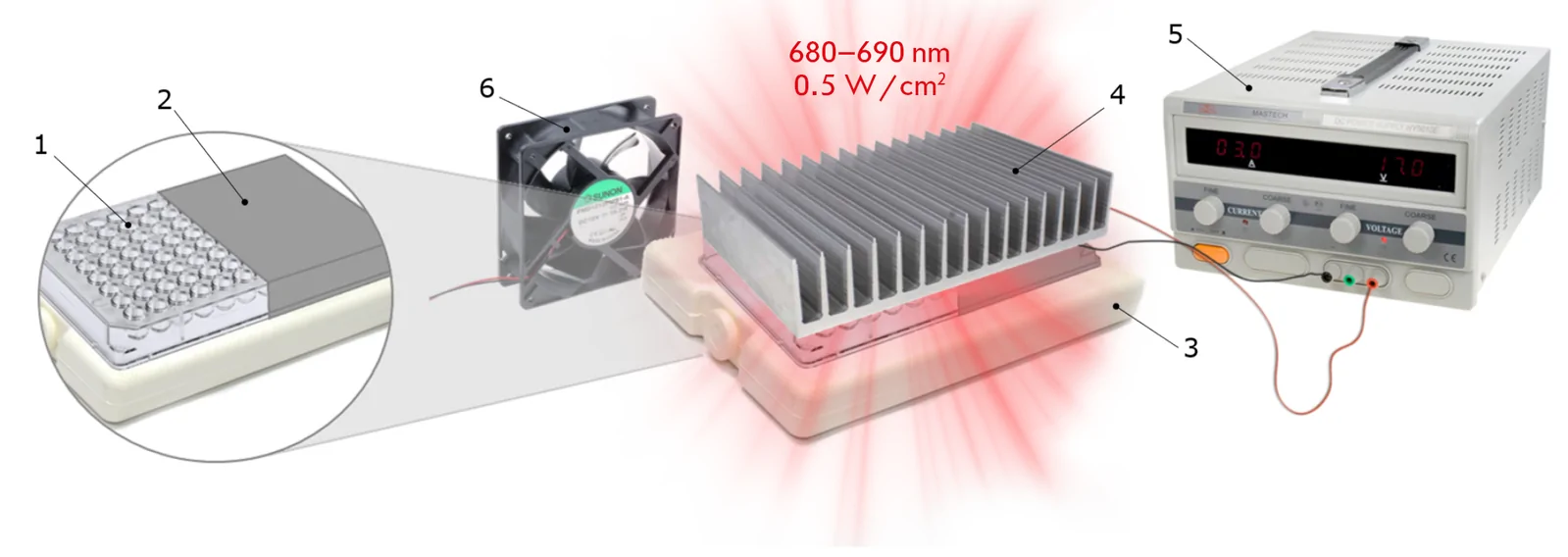
Presentation of the experimental setup used to irradiate cells with light: (*1*) 96-well plate with cells; (*2*) aluminum foil; (*3*) cooling plastic block; (*4*) diode matrix; (*5*) power supply; and (*6*) fan

A 96-well plate (SPL Life Sciences Co., Republic of Korea) containing pre-seeded cells and added test substances was placed on a cooled plastic block (5°C). One half of the plate (48 wells) was covered with aluminum foil to prevent light exposure (non-irradiated control group). LEDs with a wavelength of 680–690 nm (1 W Infrared LED, Hezhou Litlight Electronic Co., Ltd) were positioned above the plate in six rows of eight LEDs mounted on an aluminum heat sink so that each LED ended up directly above a well. The heat sink was cooled by a separately positioned fan to prevent vibration. The LEDs were connected to a constant-current power supply (0.5 A per LED); the irradiance of the LED assembly was 0.5 W/cm^2^.

**Evaluation of light exposure in cell cultures and optimization of the experimental PDT conditions**

The effect of prolonged light exposure (0–60 min) on cells was investigated using SKBR-3, BT-474, SKOV-3, and PC-3 cell lines. Twenty-four hours before the experiment, cells were seeded into 96-well plates (5 × 10^3^ cells/well for SKBR-3, SKOV-3, and PC-3; 1 × 10^4^ cells/well for BT-474). Irradiation was performed for 0–60 min. Half of each plate, with cell-containing wells covered with aluminum foil, served as a nonirradiated control. After completion of the experimental procedures, the cells were incubated for 24 h (37°C, 5% CO_2_). Cell viability after irradiation was determined using the standard MTT assay.

**Evaluation of the phototoxicity of IR700 and the DARPin G3-ABD-IR700 conjugate**

SKBR-3, SKOV-3, BT-474, and PC-3 cells were seeded into 96-well plates at 5 × 10^3^ cells/well (SKBR-3, SKOV-3, and PC-3) or 1 × 10^4^ cells/well (BT-474) and cultured for 24 h. The cells were then incubated for 60 min at 37°C in a DMEM/F-12 medium with free IR700 or DARPin G3-ABD-IR700 at specified concentrations (IR700: 0.1, 0.2, 0.3, 0.4, and 0.5 μM; DARPin G3-ABD-IR700: 0.6, 0.3, 0.06, 0.03, 0.006, and 0.003 μM). The wells were divided into groups with or without washing with phosphate-buffered saline (PBS). At the end of incubation, the medium was removed from the wells in the washed group; the cells were washed twice with 100 μL PBS, and then a 100 μL culture medium was added. In the unwashed wells, the test-substance solution in the culture medium was retained. All the procedures involving PSs were performed under dim light; plates were protected from light with aluminum foil, except during PDT. One half of each 96-well plate, containing the test samples, was irradiated with the LED array for 60 min, whereas the other half was covered with aluminum foil (non-irradiated control group). After irradiation, cells were incubated for 24 h (37°C, 5% CO_2_). Phototoxicity was assessed by the standard MTT assay.

**Evaluation of specific binding of the DARPin G3-ABD-IR700 conjugate to HER2**

Twenty-four hours before the experiment, SKBR-3 cells were seeded in 96-well plates at 5 × 10^3^ cells/well. After incubation, the medium was removed and either a 50 μL DARPin G3 solution (2 μM) to block HER2 receptors or a 50 μL culture medium was added to the cells. The cells were incubated for 30 min (37°C, 5% CO_2_). Then, a 50 μL DARPin G3-ABD-IR700 conjugate (0.4 μM) was added to each well, resulting in a final volume of 100 μL per well. The cells were incubated with the conjugate for 60 min (37°C, 5% CO_2_) and then washed twice with 100 μL PBS. The eight experimental groups included four PS-free controls (nonirradiated/no PBS wash; nonirradiated/PBS wash; irradiated/no PBS wash; and irradiated/PBS wash) and four irradiated groups containing PS and/or DARPin G3 (PS/PBS wash; PS/no PBS wash; PS + DARPin G3/PBS wash; and DARPin G3/PBS wash). The cells in non-irradiated groups remained covered with aluminum foil. After all the experimental procedures, the cells were incubated for 24 h (37°C, 5% CO_2_) and then cell viability was evaluated.

**Assessment of cell viability**

Cell viability was assessed using the standard MTT assay. Twenty-four hours after irradiation, the culture medium was removed from all the wells of the 96-well plates and replaced with a fresh medium containing a 0.5 mg/mL MTT reagent. The cells were incubated in an MTT solution for 4 h (5% CO_2_, 37°C). The MTT-containing medium was then removed from all the wells, and DMSO (100 μL/well) was added. The optical density was measured using a Multiscan FS spectrophotometer (Thermo Fisher Scientific Inc., Waltham, MA, USA). Cell viability was calculated as the ratio between the optical density of the experimental wells and that of the control wells.

**Statistical analysis**

Data were processed using the GraphPad Prism software (version 8.0.1 for Windows; GraphPad Software, USA). The Student’s t-test was used to determine significant intergroup differences (*p* < 0.05). Results are presented as a mean ± standard deviation.

## RESULTS AND DISCUSSION

The DARPin G3-ABD-IR700 conjugate was developed by nonspecific conjugation of a IRDye 700DX succinimidyl ester to lysine amino groups in the DARPin G3-ABD fusion protein. The synthesis is based on an acylation reaction. The lone electron pair on the nitrogen atom of the lysine amino group enables its attachment to the carbonyl carbon atom of the IR700 molecule. Subsequent charge redistribution within the structure results in cleavage of N-hydroxysuccinimide and formation of the reaction product, DARPin G3-ABD-IR700 (*Fig. 2*).

**Fig. 2:**
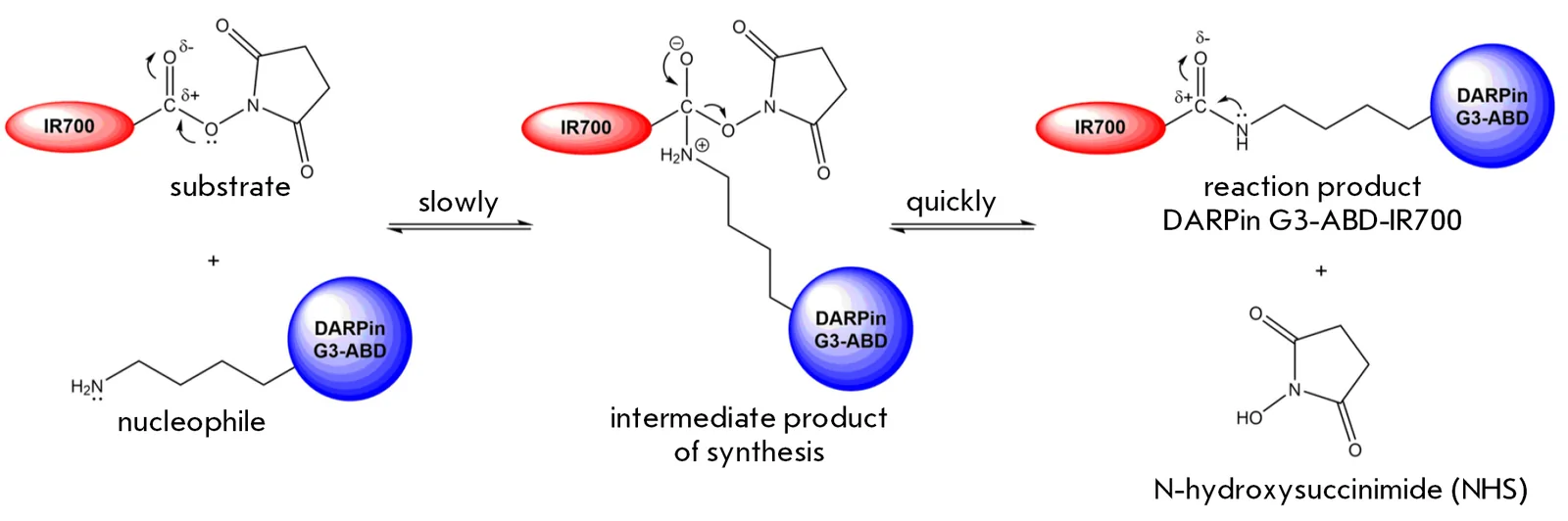
Scheme of conjugation of compound IR700 to a DARPin G3-ABD molecule

The purity of the DARPin G3-ABD-IR700 conjugate, determined by polyacrylamide gel electrophoresis, was 93 ± 3%. A homodimer impurity was present, which is characteristic of DARPin-family molecules bearing a terminal cysteine [23]. The ratio between the starting protein (DARPin G3-ABD) and the PS molecules in the conjugate was determined by spectrophotometric analysis: one protein equivalent corresponded to 1.4 IR700 equivalents.

The phototoxicity of free IR700 and DARPin G3-ABD-IR700 was investigated to assess the biological efficacy of the developed conjugate.

To optimize photoexposure parameters, an LED-array irradiation regimen was first selected and the dependence of cell-culture viability on exposure time was evaluated (*Fig. 3*). HER2-overexpressing human breast cancer SKBR-3 cells, the anticipated primary target of the developed conjugate, were selected as the main biological model. SKBR-3 cells retained high viability regardless of irradiation duration (*Fig. 3A*). Experiments with other tumor cell cultures (PC-3, SKOV-3, and BT-474) demonstrated that these cell lines are highly resistant to prolonged light exposure for 60 min (*Fig. 3B*). Thus, the applied LED irradiation conditions neither exerted an intrinsic cytotoxic effect nor affected the viability of the studied cell cultures.

**Fig. 3:**
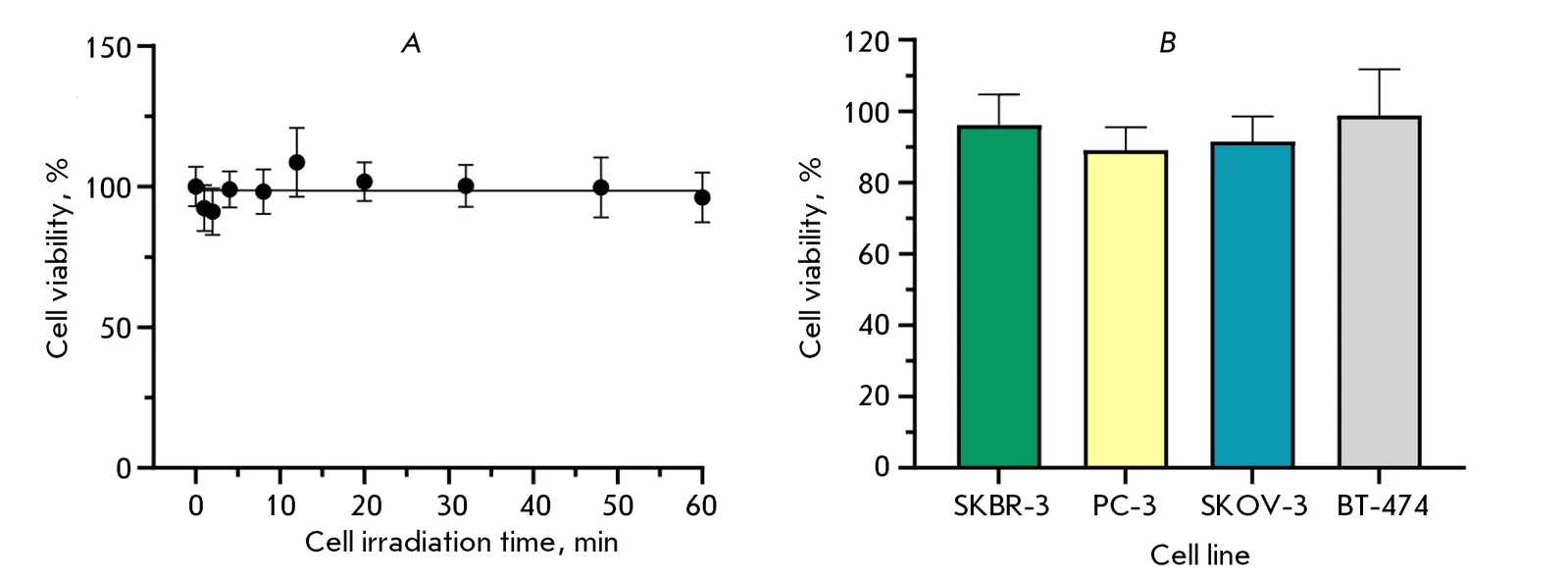
The results of the study of the effect of irradiation time on the viability of SKBR-3 cells (*A*) and prolonged exposure to light (for 60 min) on the SKBR-3, SKOV-3, BT-474 and PC-3 cell lines (*B*)

The suitability of the 60-min irradiation period was confirmed by preliminary phototoxicity tests with IR700 at concentrations of 1.0 and 0.2 μM in SKBR-3 cells after 30 and 60 min of light exposure (*Fig. 4*). At the initial stage of the study, test-solution concentrations were selected based on an analysis of published data [27].

**Fig. 4:**
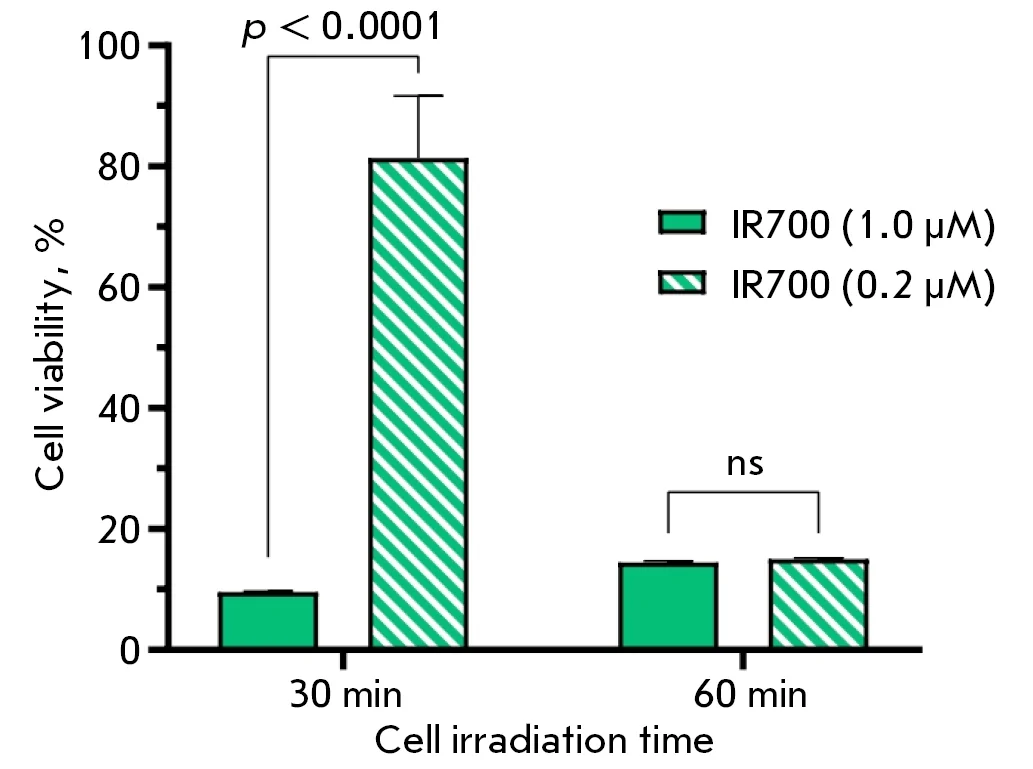
The results of primary tests to assess the phototoxicity of IR700 (1.0 and 0.2 μM) on the SKBR-3 cell line after irradiation with diode light for 30 and 60 min (ns – no statistically significant differences)

As shown in *Fig. 4*, 30-min exposure did not provide the required phototoxicity of 0.2 μM IR700, whereas 60-min irradiation resulted in the death of more than 80% of the cells, with no statistically significant differences between concentrations. Therefore, the 60-min illumination period was standardized in subsequent experiments to ensure reproducible photodynamic activation.

*Figure 5* presents the phototoxicity data, including half-maximal inhibitory concentration (IC_50_) values, for free IR700 in SKBR-3, PC-3, SKOV-3, and BT-474 cell lines without PBS washing. BT-474 breast cancer cells were found to be more sensitive to IR700 than the other cell lines. Notably, the test compound exhibited photosensitizing properties only in the experiments without PBS washing. In the respective tests where cells were washed before irradiation, viability remained within 80–100%. Therefore, IR700 did not bind to the cells and was almost completely removed through washing.

**Fig. 5:**
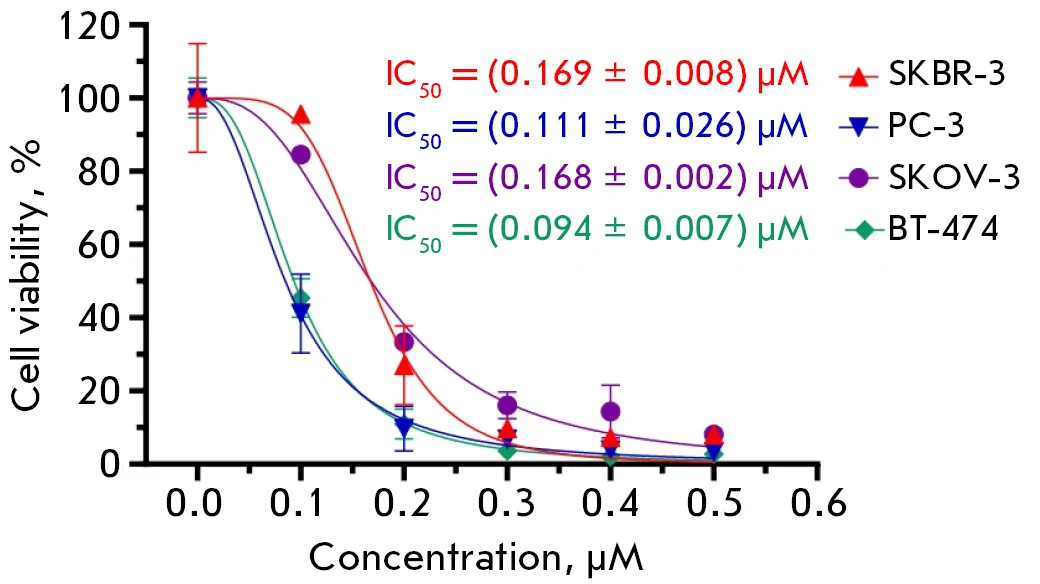
The results of the evaluation of the phototoxicity of the individual substance IR700 on the SKBR-3, PC-3, SKOV-3 and BT-474 cell lines (without PBS washing)

These data (*Fig. 5*) indicate that unconjugated IR700 mediates phototoxicity predominantly through nonspecific reversible interactions, without accumulating on the surface of target cells or within them in significant amounts, as confirmed by the fact that this effect is completely lost after PBS washing. The absence of a decline in viability after washing indicates that IR700 neither enters cells nor forms the stable receptor-mediated complexes required for selective accumulation in tumor tissue.

Unlike that for free IR700, the photosensitizing activity of DARPin G3-ABD-IR700 was retained after washing with PBS. Collectively, these data confirm that conjugation of IR700 to the targeting protein DARPin G3-ABD ensures specific binding to cellular targets and more stable membrane association, which is critical for achieving a photodynamic effect after the free photosensitizer is removed.

*Figure 6* shows the relationship between the viability of different tumor cell cultures and the concentration of DARPin G3-ABD-IR700 after washing with PBS and irradiation. PC-3 cells were characterized by the lowest sensitivity, which is consistent with low HER2 expression and substantiates the receptor-mediated mechanism of action of the conjugate.

**Fig. 6:**
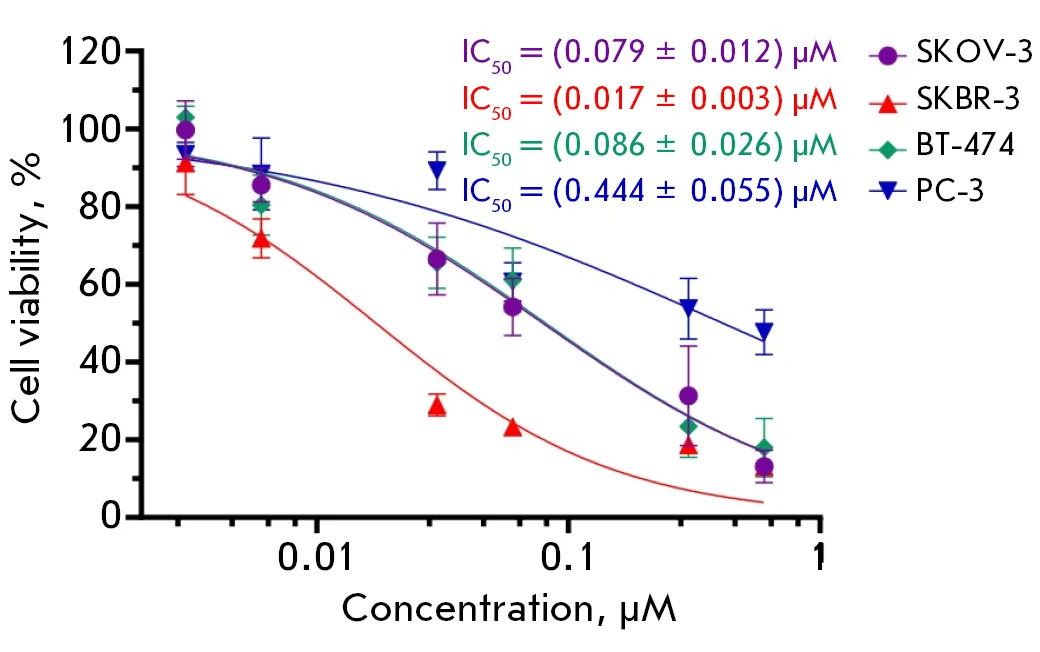
The results of the evaluation of the phototoxicity of the DARPin G3-ABD-IR700 conjugate on the SKOV-3, SKBR-3, BT-474 and PC-3 cell lines (after washing with PBS)

The IC_50_ values were calculated for IR700 and DARPin G3-ABD-IR700 based on the data obtained (*Table 1*). The developed conjugate was more effective against SKBR-3 and SKOV-3 cells than free IR700, since the IC_50_ values of IR700 were at least twofold higher than those for the conjugate. The observed differences can be attributed to the higher affinity of DARPin G3 for HER2, as well as to the variability in target expression among the studied cultures.

**Table 1:** Half-maximal inhibitory concentration (IC_50_) values for IR700 and DARPin G3-ABD-IR700 in different tumor cell cultures (*n* = 4)

Cell line	IC_50_, μM	
	IR700	DARPin G3-ABD-IR700
SKBR-3	0.169 ± 0.008	0.017 ± 0.003
SKOV-3	0.168 ± 0.002	0.079 ± 0.012
BT-474	0.094 ± 0.007	0.086 ± 0.026
PC-3	0.111 ± 0.026	0.444 ± 0.055

The binding specificity of DARPin G3-ABD-IR700 to HER2 on tumor cells was investigated in order to evaluate the ligand–receptor interaction mechanisms (*Fig. 7*). HER2-overexpressing SKBR-3 breast cancer cells were used in this experiment. Receptors were blocked using an excess of the DARPin G3 protein.

**Fig. 7:**
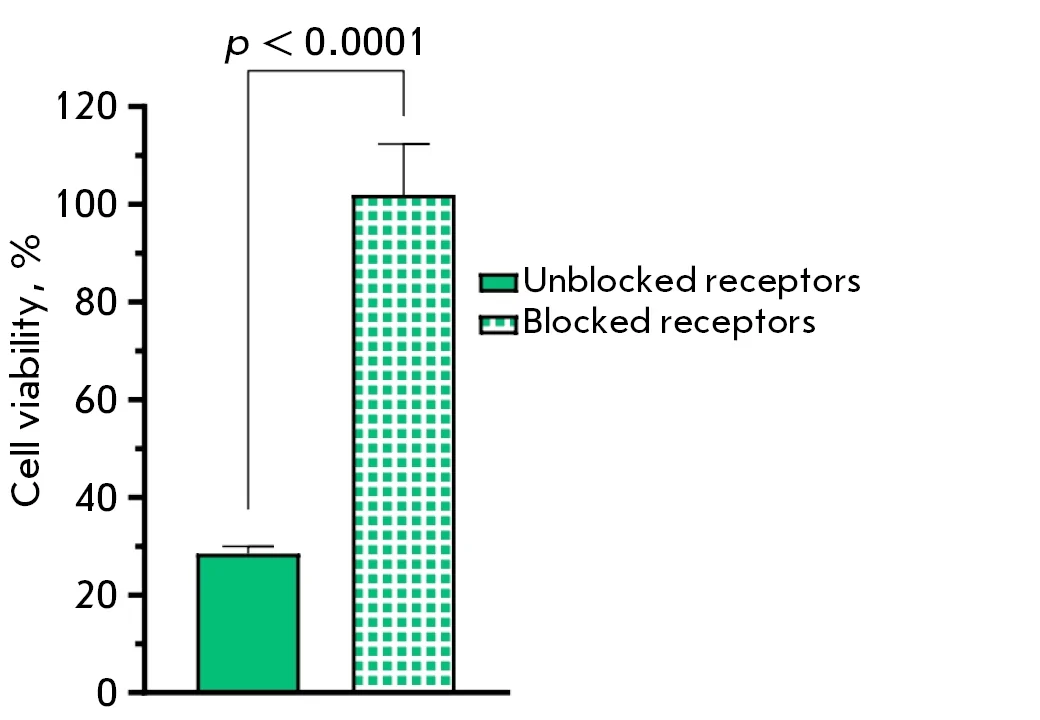
The results of the study of the binding specificity of DARPin G3-ABD-IR700 to the HER2 receptor on the SKBR-3 cell line after washing with PBS and irradiation for 60 min

Analysis of the results (*Fig. 7*) showed pronounced cell death in the group with unblocked receptors, whereas viability in the blocked group was comparable to that in the control group (100%). The statistically significant decrease in cell viability in the group with unblocked HER2 receptors compared to the pre-blocking condition attests to a receptor-mediated mechanism of action of DARPin G3-ABD-IR700 and its selective binding to HER2-positive cells, fully consistent with the concept of targeted PDT.

The principal mechanism underlying the observed phototoxic action of the developed PS is selective binding of the conjugate, through its DARPin G3-ABD protein moiety, to HER2, followed by IR700 activation by 690 nm light. As described previously [28], this causes the release of the hydrophilic axial ligands of IR700 and alters its solubility. Insoluble hydrophobic regions are formed within the conjugate molecule, thus generating physical stress at the membrane surface and leading to damage to the cell.

Hence, the developed targeted conjugate has two fundamental advantages over unconjugated IR700. First, the targeting component (DARPin G3) increases the local concentration of the photosensitizer on target–cell surfaces through high-affinity, saturable interaction with HER2, potentially lowering the dose required to achieve a cytotoxic effect. Second, selective accumulation of the conjugate on HER2-positive cells minimizes off-target binding and phototoxicity to HER2-negative cells and normal tissues, thereby improving the therapeutic index of PDT.

## CONCLUSION

A novel DARPin G3-ABD-IR700 targeted conjugate for photodynamic therapy was synthesized in this work. *In vitro* studies confirmed the high phototoxic activity of the developed conjugate against HER2-positive cells (*Fig. 8*). The conjugate demonstrates specific binding to HER2, the major therapeutic target in many cancer types, ensuring targeted delivery of the photosensitizer, followed by photodestruction of tumor cells upon irradiation. Our findings provide grounds to considser DARPin G3-ABD-IR700 a promising targeted agent for PDT of HER2-positive cancer.

**Fig. 8:**
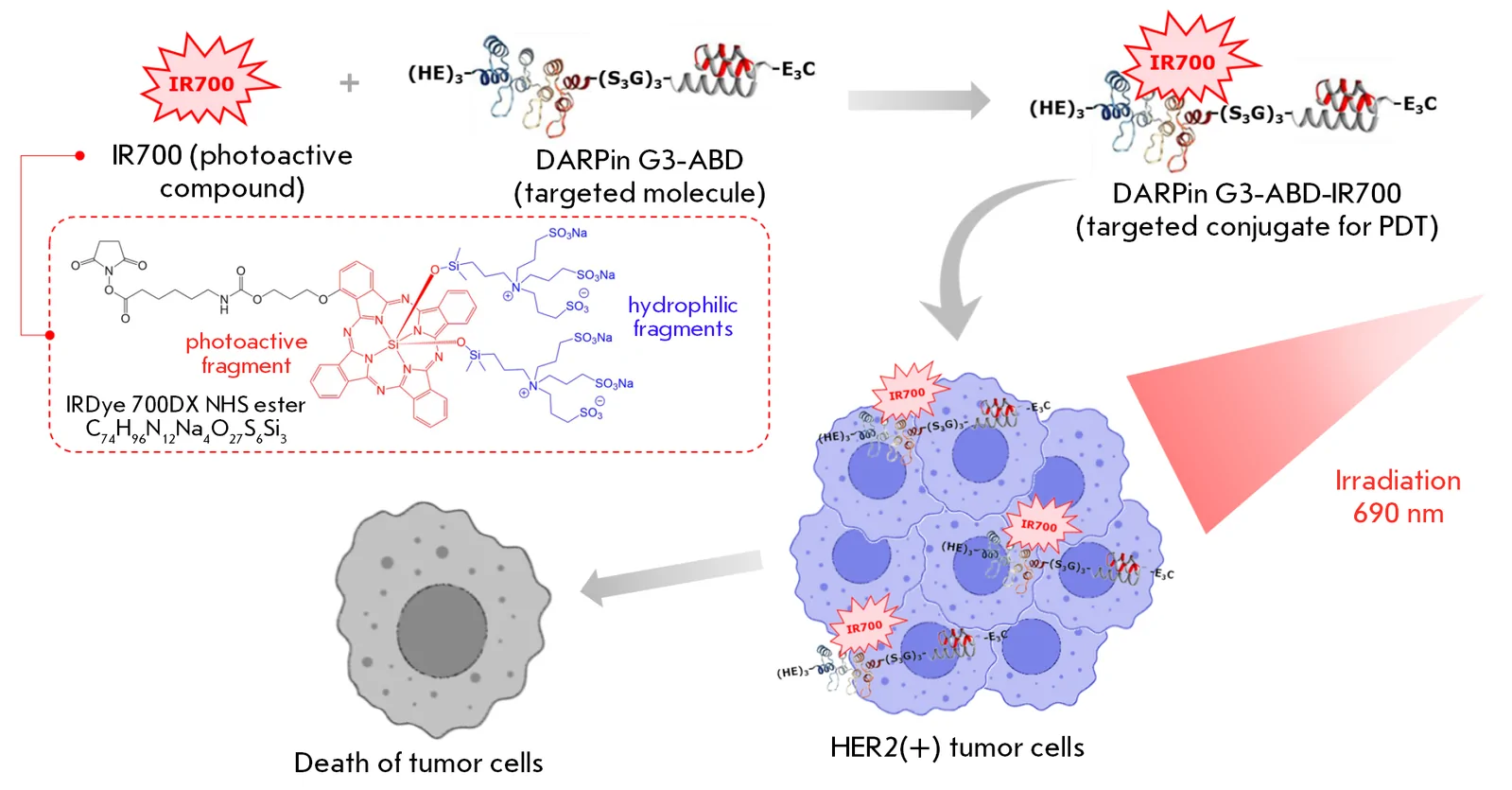
The schematic diagram of preparation and the main mechanism of antitumor action of the novel targeted photodynamic conjugate DARPin G3-ABD-IR700

## Abbreviations

PDT: photodynamic therapy
PS: photosensitizer
NIR: near-infrared
HER2: human epidermal growth factor receptor 2
DARPin: designed ankyrin repeat protein
ABD: albumin-binding domain
DMSO: dimethyl sulfoxide
PBS: phosphate-buffered saline
